# Subjective Outcome Evaluation of the Project P.A.T.H.S. (Secondary 2 Program): Views of the Program Participants

**DOI:** 10.1100/tsw.2009.119

**Published:** 2009-10-01

**Authors:** Daniel T. L. Shek, Catalina S. M. Ng

**Affiliations:** ^1^ Department of Applied Social Sciences, The Hong Kong Polytechnic University, Hong Kong, China; ^2^ Department of Sociology, East China Normal University, Shanghai, China; ^3^ Kiang Wu Nursing College of Macau, Macau, China

**Keywords:** subjective outcome evaluation, Chinese adolescents, quantitative data, positive youth development program

## Abstract

A total of 196 secondary schools participated in the Secondary 2 Program of the Full Implementation Phase of the Project P.A.T.H.S. (Positive Adolescent Training through Holistic Social Programmes). After completion of the Tier 1 Program, 30,731 students responded to the Subjective Outcome Evaluation Form (Form A) to assess their perceptions of the program, instructors, and perceived effectiveness of the program. Based on the consolidated reports submitted by the schools to the funding body, the research team aggregated the consolidated data to form a “reconstructed” overall profile on the perceptions of the program participants. Findings demonstrated that high proportions of the respondents had positive perceptions of the program and the instructors, and roughly four-fifths of the respondents regarded the program as beneficial to them. Correlation analyses showed that perceived program and instructor characteristics were positively associated with perceived benefits of the program.

## INTRODUCTION

In the wake of quality assurance, with greater pressure on service providers to measure service effectiveness and outcomes, client satisfaction has emerged as an important outcome indicator in the evaluation of human services[1]. Client perceptions have, therefore, been afforded prominence when evaluating services provided. Reviews of the existing literature indicate that using the client satisfaction approach in evaluation or subjective outcome indicators has a long history in human services in different cultures[2,3,4,5]. A subjective outcome evaluation or client satisfaction survey is commonly utilized to assess the perceived benefits of a program and the degree of satisfaction that the participants have regarding the different aspects of the program. Although there are arguments against the use of subjective outcome assessment, there is mounting evidence that subjective outcome measures were correlated with objective outcome measures[6,7,8,9,10,11,12]. As one aspect of program evaluation is about how the program can be improved, the information-rich data from the clients help to evaluate the effectiveness of the program and to effect any substantial improvements in service delivery[13].

Interest in adolescent positive youth development programs has increased markedly following reports that adolescents face a number of developmental problems, such as abuse of psychotropic substances[14], adolescent suicide[15], deliberate self-harm[16], and school violence[17] in the context of Hong Kong. Added to this, with a notable lack of positive youth development programs in the territory, there is a desperate demand for adolescent positive youth development programs[18,19,20]. Against this background, a territory-wide project entitled P.A.T.H.S. to Adulthood: A Jockey Club Youth Enhancement Scheme was initiated by The Hong Kong Jockey Club Charities Trust, with an earmarked grant of HK$400 million. "P.A.T.H.S." is an acronym for **P**ositive **A**dolescent **T**raining through **H**olistic **S**ocial Programmes. With an aim to develop a multiyear universal positive youth development program to promote holistic adolescent development in Hong Kong, a research team with researchers from five local universities was formed, with the first author being the principal investigator[21,22].

In the Project P.A.T.H.S., there are two tiers of programs (Tier 1 and Tier 2). The Tier 1 Program is a universal positive youth development program where students in Secondary 1 to 3 participate in the program, normally with 20 h of training in the school year at each grade, involving 40 teaching units. In the Tier 1 Program, 15 positive youth development constructs are embedded in the 40 teaching units. These constructs include promotion of bonding, cultivation of resilience, promotion of social competence, promotion of emotional competence, promotion of cognitive competence, promotion of behavioral competence, promotion of moral competence, cultivation of self-determination, promotion of spirituality, development of self-efficacy, development of a clear and positive identity, promotion of beliefs in the future, provision of recognition for positive behavior, provision of opportunities for prosocial involvement, and fostering prosocial norms[20].

Since the Project P.A.T.H.S. is a huge project in terms of financial and manpower resources, as well as the number of participating schools in the territory, program evaluation is of paramount importance for several reasons. First, it is imperative to prove to the program funder and the Government that the project is of great benefit to students. Second, program implementers, particularly teachers and social workers, are only motivated to teach the program that is found to be effective. Finally, reviews of the literature indicate that there is a pressing need to accumulate research findings on the effectiveness of psychosocial intervention programs. For instance, in the Western context, among the 77 programs under review, only approximately one-third of them were effective[23], whereas in the Chinese context, Shek et al.[24] highlighted that evidence-based social work practice was very weak in Hong Kong.

To provide a comprehensive picture pertaining to the effectiveness of the project, numerous evaluation strategies, including objective outcome evaluation utilizing a randomized group trial[25]; subjective outcome evaluation based on quantitative and qualitative data collected from the program participants and instructors[26,27]; qualitative evaluation based on focus groups involving students and instructors[28,29]; in-depth interviews with program implementers, student logs, and student products[30]; process evaluation involving systematic observations of delivery of the program[31]; and interim evaluation[32] are employed. The aforementioned mechanisms consistently provide strong evidence that the Project P.A.T.H.S. has a beneficial influence on students[33,34,35].

With specific reference to subjective outcome evaluation, quantitative findings based on the Secondary 2 Program of the Experimental Implementation Phase[36] demonstrated that program participants (n =7,406 students from 49 schools) in the academic year 2006/07 perceived the program positively, including the clear objectives of the curriculum (79.4%), systematic planning of activities (77.9%), peer interaction among students (77.5%), and active involvement of students during class (76.6%). Moreover, a high proportion of the students (84.9%) had positive evaluation of the instructors. About 86% of the respondents responded that the instructor was very involved and approximately 85% indicated that the instructor was well prepared for the lessons, encouraged students to participate in the activities, and was ready to provide assistance to students in need. Further, about four-fifths of the respondents perceived that the program enhanced their overall development, including the ability to resist harmful influences (79.4%), ability to distinguish between the good and the bad (81.4%), competence in making sensible and wise choices (79.7%), and compassion and caring about others (79.3%). Overall, about 80% of the participants were satisfied with the course and more than 70% of the participants would recommend the program to their friends who have similar needs.

As the preceding sections illustrate, evidence from different evaluation strategies supported the effectiveness of the Tier 1 Program of the Project P.A.T.H.S. It is imperative to examine whether such findings can be replicated across time and participants. In the current study, the data collected from 196 schools that joined the Secondary 2 Program of the Full Implementation Phase were examined. As the Project P.A.T.H.S. was financially supported by The Hong Kong Jockey Club Charities Trust, each participating school had to submit an evaluation report with the consolidated subjective outcome evaluation profile of the school to the funding body. Such reports were then used by the research team to "reconstruct" the overall profile of the subjective outcome evaluation data. The major advantage of this strategy is to promote evaluation in the field and to conduct secondary data analyses of the reports submitted concurrently.

## METHODS

### Participants and Procedures

A total of 196 schools participated in the second year of the Full Implementation Phase in the Secondary 2 level in the academic year 2007/08. The mean number of students per school was 170.66 (range: 12–280), with an average of 4.62 classes per school (range: 1–8). Among them, 83 schools adopted the full program (i.e., 20-h program involving 40 units), while 113 schools adopted the core program (i.e., 10-h program involving 20 units). The mean number of sessions used to implement the program was 22.81 (range: 7–60). While 108 (55.10%) schools incorporated the program into the formal curriculum (e.g., Liberal Studies, Life Education), 88 schools (44.90%) used other modes of implementation (e.g., form teacher periods and other combinations). The mean number of social workers and teachers implementing the program per school were 1.97 (range: 0–8) and 5.59 (range: 0–15), respectively.

After the completion of the Tier 1 Program, the participants were invited to respond to the subjective outcome evaluation questionnaire (Form A), which was developed by the research team. A total of 30,731 students (mean =156.80 students per school, range: 12–243) responded to Form A. The data collection was normally conducted at the last session of the program. On the day when the evaluation data were collected, the purpose of the evaluation was explained and the confidentiality of the data collected was repeatedly emphasized to all students. The students were asked to indicate their wish if they did not want to respond to the evaluation questionnaire (i.e., "passive" informed consent was obtained from the students). All participants responded to all scales in the evaluation form in a self-administration format. Adequate time was provided for the participants to complete the questionnaire. To facilitate the program evaluation, the research team developed an evaluation manual with standardized instructions for collecting the subjective outcome evaluation data[37]. In addition, adequate training was provided to the workers during the 20-h training workshops on how to collect and analyze the data collected by Form A.

### Instruments

The Subjective Outcome Evaluation Form (Form A) designed by Shek and Siu[37] consists of the following parts:

- Participants' perceptions of the program, such as program objectives, design, classroom atmosphere, interaction among the students, and the respondents' participation during class (10 items)

- Participants' perceptions of the workers, such as the preparation of the instructor, professional attitude, involvement, and interaction with the students (10 items)
- Participants' perceptions of the effectiveness of the program, such as promotion of different psychosocial competencies, resilience, and overall personal development (16 items)
- The extent to which the participants would recommend the program to other people with similar needs (1 item)
- The extent to which the participants would join similar programs in future (1 item)
- Overall satisfaction with the program (1 item)
- Things that the participants learned from the program (open-ended question)
- Things that the participants appreciated most (open-ended question)
- Opinion about the instructor(s) (open-ended question)
- Areas that require improvement (open-ended question)

For the closed-ended questions, the workers who collected the data were requested to input the data in an EXCEL file developed by the research team that would automatically compute the frequencies and percentages associated with the different ratings for an item. When the schools submitted the reports, they were also requested to submit the soft copy of the consolidated data sheets. In the reports prepared by the schools, the workers were also required to estimate the degree of adherence to the program manuals (i.e., the extent to which the program was implemented in accordance with the program manuals). After receiving the consolidated data from the funding body, the research team aggregated the data to "reconstruct" the overall profile based on the subjective outcome evaluation data.

## RESULTS

Reliability analyses with the schools as the unit of analyses demonstrated that Form A has relatively high internal consistency: 10 items related to the program ($\alpha = 0.98$, mean interitem correlation = 0.86), 10 items related to the instructor ($\alpha = 0.99$, mean interitem correlation = 0.94), 16 items related to the benefits ($\alpha = 0.99$, mean interitem correlation = 0.93), and 39 items based on the entire Form A ($\alpha = 0.99$, mean interitem correlation = 0.81).

Based on the closed-ended questions, several salient findings can be highlighted. First, roughly three-quarters of the respondents perceived the program in a positive manner (Table 1). For instance, 82.26% of the students indicated that the program objectives were very clear and 81.24% felt that there was much peer interaction in the program. Second, a high proportion of the students had positive evaluation of the instructors (Table 2) as 87.68% of the respondents showed that the instructors were ready to provide assistance to them when needed and 87.58% of the respondents found that the instructors were very involved. Third, as shown in Table 3, roughly four-fifths of the respondents perceived that the program promoted their development, including social competence (80.92%), ability to resist harmful influences (81.16%), moral competence (82.72%), ability in making sensible and wise choices (81.89%), and overall development (81.74%). Fourth, while roughly three-quarters of the participants would recommend the program to their friends with similar needs, only a simple majority of them (62.79%) would join similar programs in the future (Table 4). Finally, roughly four-fifths of the respondents demonstrated that they were satisfied with the program (Table 4). Regarding the degree of program adherence estimated by the workers, the mean level of adherence was 86.63%, with a range from 34 to 100%.

The total scores based on items on perceived program characteristics were positively correlated with different measures of perceived benefits of the program. Similarly, the total scores based on items on perceived instructor characteristics were positively correlated with different measures of perceived benefits of the program. Table 5 shows the related correlation coefficients.

**TABLE 1**
**Summary of the Views of the Program Participants on the Program**

| | Strongly Disagree (1) | | Disagree (2) | | Slightly Disagree (3) | | Slightly Agree (4) | | Agree (5) | | Strongly Agree (6) | | Participants with Positive Response (Options 4–6) | |
|---|---|---|---|---|---|---|---|---|---|---|---|---|---|---|
| | n | % | n | % | n | % | n | % | n | % | n | % | n | % |
| The objectives of the curriculum are very clear (n = 30,504) | 977 | 3.20 | 1125 | 3.69 | 3308 | 10.84 | 10,318 | 33.83 | 11,675 | 38.27 | 3101 | 10.17 | 25,094 | 82.26 |
| The design of the curriculum is very good (n = 30,468) | 1011 | 3.32 | 1511 | 4.96 | 4073 | 13.37 | 11,442 | 37.55 | 9983 | 32.77 | 2448 | 8.03 | 23,873 | 78.35 |
| The activities were carefully planned (n = 30,429) | 936 | 3.08 | 1249 | 4.10 | 3680 | 12.09 | 11,163 | 36.69 | 10,727 | 35.25 | 2674 | 8.79 | 24,564 | 80.73 |
| The classroom atmosphere was very pleasant (n = 30,370) | 1193 | 3.93 | 1471 | 4.84 | 3943 | 12.98 | 9673 | 31.85 | 9822 | 32.34 | 4268 | 14.05 | 23,763 | 78.24 |
| There was much peer interaction among the students (n = 30,272) | 992 | 3.28 | 1193 | 3.94 | 3494 | 11.54 | 9485 | 31.33 | 10,399 | 34.35 | 4709 | 15.56 | 24,593 | 81.24 |
| I participated actively during lessons (including discussions, sharing, games, etc.) (n = 30,413) | 1059 | 3.48 | 1322 | 4.35 | 3676 | 12.09 | 10416 | 34.25 | 9824 | 32.30 | 4116 | 13.53 | 24,356 | 80.08 |
| I was encouraged to do my best (n = 30,423) | 1250 | 4.11 | 1614 | 5.31 | 4565 | 15.01 | 11,226 | 36.90 | 8933 | 29.36 | 2835 | 9.32 | 22,994 | 75.58 |
| The learning experience I encountered enhanced my interest towards the lessons (n = 30,341) | 1409 | 4.64 | 1718 | 5.66 | 4380 | 14.44 | 10,865 | 35.81 | 9230 | 30.42 | 2739 | 9.03 | 22,834 | 75.26 |
| Overall, I have very positive evaluation of the program (n = 30,393) | 1414 | 4.65 | 1810 | 5.96 | 4402 | 14.48 | 10,596 | 34.86 | 9256 | 30.45 | 2915 | 9.59 | 22,767 | 74.91 |
| On the whole, I like this curriculum very much (n = 30,343) | 1781 | 5.87 | 1767 | 5.82 | 4076 | 13.43 | 9866 | 32.51 | 9246 | 30.47 | 3607 | 11.89 | 22,719 | 74.87 |

## DISCUSSION

This paper solely reports the quantitative evaluation findings based on the closed-ended questions, whereas qualitative findings based on the open-ended questions would be presented elsewhere. The findings of this study provide additional support for the effectiveness of the Project P.A.T.H.S. among junior secondary school students in Hong Kong as results suggest that a large majority of respondents perceived both the program and workers positively, and approximately four-fifths of the respondents regarded the program to be beneficial to their development. Congruent with the findings from previous studies on the program, the Project P.A.T.H.S. was rated very highly by program participants. Overall, clients are satisfied with the program.

However, despite the fact that most of the participants had positive perceptions of the program and the workers, and perceived the program to be effective, as well as about three-quarters of the participants who claimed that they would recommend the program to peers with similar needs, contrary to our expectations, only 62.79% of the participants responded that they would join similar programs in the future. To unfold the puzzle, further analyses of the qualitative data are needed.

**TABLE 2**
**Summary of the Views of the Program Participants of the Workers Implementing the Program**

| | Strongly Disagree (1) | | Disagree (2) | | Slightly Disagree (3) | | Slightly Agree (4) | | Agree (5) | | Strongly Agree (6) | | Participants with Positive Responses (Options 4–6) | |
|---|---|---|---|---|---|---|---|---|---|---|---|---|---|---|
| | n | % | n | % | n | % | n | % | n | % | n | % | n | % |
| The instructor(s) had a good mastery of the curriculum (n = 30,290) | 771 | 2.55 | 958 | 3.16 | 2631 | 8.69 | 9430 | 31.13 | 11,958 | 39.48 | 4542 | 15.00 | 25,930 | 85.61 |
| The instructor(s) was well prepared for the lessons (n = 30,274) | 731 | 2.41 | 839 | 2.77 | 2283 | 7.54 | 8909 | 29.43 | 12,315 | 40.68 | 5197 | 17.17 | 26,421 | 87.27 |
| The instructor(s)' teaching skills were good (n = 30,248) | 837 | 2.77 | 935 | 3.09 | 2912 | 9.63 | 9207 | 30.44 | 11,732 | 38.79 | 4625 | 15.29 | 25,564 | 84.51 |
| The instructor(s) showed good professional attitudes (n = 30,216) | 812 | 2.69 | 857 | 2.84 | 2345 | 7.76 | 8802 | 29.13 | 11,997 | 39.70 | 5403 | 17.88 | 26,202 | 86.72 |
| The instructor(s) was very involved (n = 30,243) | 788 | 2.61 | 778 | 2.57 | 2191 | 7.24 | 8370 | 27.68 | 12,123 | 40.09 | 5993 | 19.82 | 26,486 | 87.58 |
| The instructor(s) encouraged students to participate in the activities (n = 30,239) | 791 | 2.62 | 751 | 2.48 | 2273 | 7.52 | 8460 | 27.98 | 12,117 | 40.07 | 5847 | 19.34 | 26,424 | 87.38 |
| The instructor(s) cared for the students (n = 30,247) | 887 | 2.93 | 899 | 2.97 | 2632 | 8.70 | 8944 | 29.57 | 11,603 | 38.36 | 5282 | 17.46 | 25,829 | 85.39 |
| The instructor(s) was ready to offer help to students when needed (n = 30,225) | 817 | 2.70 | 745 | 2.46 | 2163 | 7.16 | 8673 | 28.69 | 12,125 | 40.12 | 5702 | 18.87 | 26,500 | 87.68 |
| The instructor(s) had much interaction with the students (n = 30,252) | 902 | 2.98 | 1008 | 3.33 | 3054 | 10.10 | 9274 | 30.66 | 11,124 | 36.77 | 4890 | 16.16 | 25,288 | 83.59 |
| Overall, I have very positive evaluation of the instructors (n = 30,288) | 938 | 3.10 | 810 | 2.67 | 2016 | 6.66 | 8211 | 27.11 | 12,247 | 40.44 | 6066 | 20.03 | 26,524 | 87.57 |

As expected, evidence suggested that there was a substantial correlation between perceived program characteristics and items on perceived benefits of the program. Results provide clear insights of how participants perceived the program is associated with the perceived benefits of the program. Clear program objectives, carefully planned activities, meticulous design of the curriculum, pleasant classroom atmosphere, and high peer interaction are all related to the quality of program implementation. With all these program features, it is more likely to bring positive program outcomes and have beneficial effects on program participants. Further, the examination of correlates of items on how participants perceived instructors was significantly associated with different measures of perceived benefits of the program. Evidently, the characteristics of effective or highly rated workers could be reflected by both (1) the professionalism of the workers, in terms of his/her knowledge of the curriculum, good teaching skills, and preparation for the lessons; and (2) his/her personal qualities of the workers, such as caring, encouraging, and being helpful as reflected by the items on the characteristics of instructors. These findings offer a few glimpses of what the important features of the program and characteristics of instructors may be. Of course, one alternative explanation for the high correlation among the different domains may be due to common method variance.

**TABLE 3**
**Perceived Effectiveness of the Program by the Program Participants**

| The extent to which the course (i.e., the program that all students joined) helped you (n = No. of responses) | Unhelpful (1) | | Not Very Helpful (2) | | Slightly Helpful (3) | | Helpful (4) | | Very Helpful (5) | | Participants with Positive Responses (Options 3–5) | |
|---|---|---|---|---|---|---|---|---|---|---|---|---|
| | n | % | n | % | n | % | n | % | n | % | n | % |
| It has strengthened my bonding with teachers, classmates, and family (n = 30,408) | 2011 | 6.61 | 5357 | 17.62 | 12,399 | 40.78 | 8400 | 27.62 | 2241 | 7.37 | 23,040 | 75.77 |
| It has strengthened my resilience in adverse conditions (n = 30,415) | 1731 | 5.69 | 4962 | 16.31 | 11,619 | 38.20 | 9384 | 30.85 | 2719 | 8.94 | 23,722 | 77.99 |
| It has enhanced my social competence (n = 30,346) | 1594 | 5.25 | 4195 | 13.82 | 11,583 | 38.17 | 9812 | 32.33 | 3162 | 10.42 | 24,557 | 80.92 |
| It has improved my ability in handling and expressing my emotions (n = 30,358) | 1652 | 5.44 | 4422 | 14.57 | 11,381 | 37.49 | 9724 | 32.03 | 3179 | 10.47 | 24,284 | 79.99 |
| It has enhanced my cognitive competence (n = 30,350) | 1696 | 5.59 | 4512 | 14.87 | 11,382 | 37.50 | 9540 | 31.43 | 3220 | 10.61 | 24,142 | 79.55 |
| My ability to resist harmful influences has been improved (n = 30,360) | 1592 | 5.24 | 4127 | 13.59 | 11,174 | 36.81 | 9819 | 32.34 | 3648 | 12.02 | 24,641 | 81.16 |
| It has strengthened my ability to distinguish between the good and the bad (n = 30,341) | 1492 | 4.92 | 3750 | 12.36 | 11,034 | 36.37 | 10258 | 33.81 | 3807 | 12.55 | 25,099 | 82.72 |
| It has increased my competence in making sensible and wise choices (n = 30,342) | 1544 | 5.09 | 3951 | 13.02 | 11,056 | 36.44 | 10086 | 33.24 | 3705 | 12.21 | 24,847 | 81.89 |
| It has helped me to have life reflections (n = 30,345) | 1978 | 6.52 | 4270 | 14.07 | 10,485 | 34.55 | 9496 | 31.29 | 4116 | 13.56 | 24,097 | 79.41 |
| It has reinforced my self-confidence (n = 30,346) | 2065 | 6.80 | 4843 | 15.96 | 10,738 | 35.39 | 9150 | 30.15 | 3550 | 11.70 | 23,438 | 77.24 |
| It has increased my self-awareness (n = 30,345) | 1946 | 6.41 | 4516 | 14.88 | 10,851 | 35.76 | 9454 | 31.16 | 3578 | 11.79 | 23,883 | 78.70 |
| It has helped me to face the future with a positive attitude (n = 30,326) | 1797 | 5.93 | 4338 | 14.30 | 10,829 | 35.71 | 9709 | 32.02 | 3653 | 12.05 | 24,191 | 79.77 |
| It has helped me to cultivate compassion and caring about others (n = 30,336) | 1843 | 6.08 | 4074 | 13.43 | 10,980 | 36.19 | 9741 | 32.11 | 3698 | 12.19 | 24,419 | 80.50 |
| It has encouraged me to care about the community (n = 30,346) | 2027 | 6.68 | 4661 | 15.36 | 10,969 | 36.15 | 9246 | 30.47 | 3443 | 11.35 | 23,658 | 77.96 |
| It has promoted my sense of responsibility in serving the society (n = 30,352) | 1900 | 6.26 | 4491 | 14.80 | 11,019 | 36.30 | 9419 | 31.03 | 3523 | 11.61 | 23,961 | 78.94 |
| It has enriched my overall development. (n = 30,351) | 1789 | 5.89 | 3752 | 12.36 | 10,442 | 34.40 | 9907 | 32.64 | 4461 | 14.70 | 24,810 | 81.74 |

Of note is our finding that concerns the degree of program adherence in this study. The mean estimated degree of adherence in the present study (86.63%) was highly comparable to the previous findings on program adherence of the Project P.A.T.H.S.[38]. The estimated level of program adherence was relatively high in this study. In contrast, in the limited Western studies on the quality of program implementation, studies generally demonstrated that the degree of program adherence was not high. For instance, Ringwalt et al.[39] reported that one-fifth of the workers implementing the program did not utilize the curriculum guide and only 15% of them followed it very closely. It is probable that the training provided to the workers as well as the commitment of the workers (as reflected by the perceptions of the students) contributed to this high level of program adherence. This finding lends further support to the notion that it is not essential to make much modification for the Tier 1 Program among different adolescent populations. Furthermore, despite the fidelity and adaptation debate, the finding also has pointed to the importance of maintaining a high degree of program fidelity[40] so as to ensure the effectiveness of the program.

**TABLE 4**
**Other Aspects of Subjective Outcome Evaluation**

If your friends have needs and conditions similar to yours, will you suggest him/her to join this course? (n = 30,180)

| Definitely Will Not Suggest (1) | | Will Not Suggest (2) | | Will Suggest (3) | | Definitely Will Suggest (4) | | Participants with Positive Responses (Options 3–4) | |
|---|---|---|---|---|---|---|---|---|---|
| n | % | n | % | n | % | n | % | n | % |
| 2522 | 8.36 | 5257 | 17.42 | 18,388 | 60.93 | 4013 | 13.30 | 22,401 | 74.22 |

Will you participate in similar courses again in the future? (n = 30,148)

| Definitely Will Not Participate (1) | | Will Not Participate (2) | | Will Participate (3) | | Definitely Will Participate (4) | | Participants with Positive Responses (Options 3–4) | |
|---|---|---|---|---|---|---|---|---|---|
| n | % | n | % | n | % | n | % | n | % |
| 3551 | 11.78 | 7666 | 25.43 | 15,351 | 50.92 | 3580 | 11.87 | 18,931 | 62.79 |

On the whole, are you satisfied with this course? (n = 30,174)

| Very Dissatisfied (1) | | Moderately Dissatisfied (2) | | Slightly Dissatisfied (3) | | Satisfied (4) | | Moderately Satisfied (5) | | Very Satisfied (6) | | Participants with Positive Responses (Options 4–6) | |
|---|---|---|---|---|---|---|---|---|---|---|---|---|---|
| n | % | n | % | n | % | n | % | n | % | n | % | n | % |
| 1369 | 4.54 | 1402 | 4.65 | 2364 | 7.83 | 13,848 | 45.89 | 7847 | 26.01 | 3344 | 11.08 | 25,039 | 82.98 |

There are three strengths of this study. First, results are based on a huge sample size (n = 33,449 students involving 196 schools). With such a large and demographically representative sample size, this attribute substantially enhances the generalizability of the research findings to other student populations. Second, different aspects of subjective outcome, including perspectives on the program, workers, perceived effectiveness, and overall satisfaction were covered in the study. Third, the present study demonstrates the strategy of "reconstructing" the overall profile of the subjective outcomes based on the reports submitted by the participating schools. In this regard, the current study is the first published scientific study utilizing this "reconstruction" approach based on such a large number of students in the Chinese culture.

Nevertheless, although the present study contributes to the understanding of the perspectives of program participants on the Project P.A.T.H.S., it also suffers from several limitations. First, since the data were reconstructed from the reports submitted by the participating schools, the unit of analysis was the schools, rather than the individual program participants. While this limitation did not affect the reconstruction of the percentage and rating data, it will adversely affect future analyses involving relationships among variables (e.g., relationship between perception of the program and perceived program effectiveness) because only 196 rather than 30,731 units of analyses were involved. This would

**TABLE 5**
**Correlation Coefficients on the Relationships between Perceived Program Characteristics, Perceived Instructor Characteristics, and Perceived Benefits of the Program**

| | Item 1 | Item 2 | Item 3 | Item 4 | Item 5 | Item 6 | Item 7 | Item 8 |
|---|---|---|---|---|---|---|---|---|
| Program Score | 0.853** | 0.845** | 0.883** | 0.860** | 0.852** | 0.872** | 0.877** | 0.866** |
| Instructor Score | 0.654** | 0.693** | 0.722** | 0.701** | 0.707** | 0.713** | 0.754** | 0.717** |

| | Item 9 | Item 10 | Item 11 | Item 12 | Item 13 | Item 14 | Item 15 | Item 16 |
|---|---|---|---|---|---|---|---|---|
| Program Score | 0.869** | 0.832** | 0.856** | 0.859** | 0.850** | 0.814** | 0.828** | 0.867** |
| Instructor Score | 0.717** | 0.651** | 0.681** | 0.684** | 0.687** | 0.628** | 0.647** | 0.728** |

** $p < 0.01$.

*Note*: Item 1: strengthening bonding; Item 2: strengthening resilience; Item 3: strengthening social competence; Item 4: strengthening emotional competence; Item 5: strengthening cognitive competence; Item 6: strengthening behavioral competence; Item 7: strengthening moral competence; Item 8: promoting self-determination; Item 9: strengthening spirituality; Item 10: strengthening self-efficacy; Item 11: fostering positive identity; Item 12: promoting beliefs in the future; Item 13: promoting positive behaviors; Item 14: strengthening prosocial involvement; Item 15: fostering prosocial norms; Item 16: enriching overall development.

substantially reduce the power of statistical analyses. A second caveat is, while the present findings are interpreted in terms of the positive program effects and experiences of the program participants, we need to exercise caution as there are several alternative explanations. The first alternative explanation is that the students were afraid that they would be punished by the workers if they did not respond in the favorable direction. Nevertheless, this alternative explanation can be partially dismissed because the students responded anonymously. Another possible explanation of the positive findings is that the students consciously acted in a “nice” manner so as to help the workers to illustrate positive program effects. Yet, this alternative explanation could be partially dismissed because negative ratings were recorded (e.g., whether the participants would join the program again) and the students responded in an anonymous manner. The third explanation is that the high proportion of positive responses is, in fact, random responses (i.e., the students did not respond seriously). This explanation can also be dismissed because, as shown by reliability analyses, the entire scale was internally consistent.

In sum, despite these limitations, the findings of this study lend further support to the effectiveness of the Tier 1 Program and most importantly, program participants perceived the program to be beneficial to their development. These positive findings are crucial because they suggest that the program can successfully engage the students in the program implementation process. From a program evaluation perspective, as systematic evaluation of social services is still in its infancy in different Chinese contexts, this paper constitutes a model based on which future subjective outcome evaluation studies can be conducted[24,41]. Since client satisfaction is crucial in program implementation, we are continuing to carry out research on the quality of the program and to work with program participants to use the results productively to improve and maintain the high quality of the program.

## ACKNOWLEDGMENTS

The preparation for this paper and the Project P.A.T.H.S. were financially supported by The Hong Kong Jockey Club Charities Trust.
